# Management of Central Venous Stenoses and Occlusions

**DOI:** 10.1007/s00270-023-03461-7

**Published:** 2023-07-17

**Authors:** Panagiotis Kitrou, Konstantinos Katsanos, Dimitrios Karnabatidis

**Affiliations:** 1 Interventional Radiology, Patras University Hospital, Patras, Greece; 2 Medical School, University of Patras, Patras, Greece

**Keywords:** Central venous stenosis, Sharp recanalization, Covered stents, Paclitaxel-coated balloons, Vascular access, Dialysis

## Abstract

Symptomatic central venous stenosis and occlusion remains the gordian knot of vascular access. Advances in techniques, like sharp recanalization, allowed for improved success rates in crossing these difficult lesions. There is also increasing evidence of new devices in treating central venous stenosis and, at the same time, improving the time needed between interventions. High-pressure balloons, paclitaxel-coated balloons, bare metal stents and covered stents have been tested with an aim to offer additional treatment options, although obstacles still exist. In the current review, authors describe relevant techniques and options, provide the evidence and evaluate the actual implementation of these devices in this demanding field.

## Introduction

### Definition, Etiology, Symptomatology

The subclavian vein, the brachiocephalic vein and the superior vena cava are the central veins of the upper part of the body. They constitute an integral component of hemodialysis as they are the last part of the vascular access circuit before entering the heart on one hand, and the vessels for dialysis catheter insertion on the other. These are also the main reasons leading to their stenosis [1, 2]. Long-term central venous catheter insertion produces central venous stenosis (CVS), mainly due to the presence of the intravascular fibrin sheath formed around the catheter. The direct contact of the catheter and the multiple curves makes the left internal jugular catheter insertion more susceptible to stenosis compared to other sites [3]. Central venous stenosis is also attributed to the insertion of other foreign devices, such as peripherally inserted central catheters, ports and cardiac-rhythm-related devices [2]. The hemodynamic changes due to increased blood flow, and the stress caused by the ipsilateral presence of a vascular access on the other hand, also contribute to the process of stenosis by producing inflammation, endothelial trauma and finally fibrosis to the vascular wall [4]. Nonetheless, the exact mechanism and cascade of events is not yet fully known [5].

Central venous stenosis may fare a silent asymptomatic path until clinical symptoms are manifested or inadequate dialysis is performed. Hence, the incidence of CVS or occlusion (CVO) is not known as only symptomatic patients, including those with a dysfunctional vascular access, will receive venography imaging (endovascular or cross-sectional). In a study by Adwaney et al., in a population of 2811 patients, 120 were diagnosed with CVS (4.3%) [5]. In a study by Trerotola et al. who retrospectively evaluated the fistulograms of 469 patients with arteriovenous fistulas (235) and grafts (234), 51% had a CVS (119/235 and 118/237, respectively). The incidence of a CVS being symptomatic was significantly higher in grafts (29% vs. 51%, *p* = 0.0005) [6].

Clinical symptoms owed to CVS/CVO are the decisive prerequisite for treatment followed by imaging verification [2]. The main clinical symptoms are listed in Table 1. There is a correlation between the clinical symptomatology and the site of stenosis/obstruction. More specifically, symptoms from the ipsilateral arm and breast suggest a subclavian lesion; the additional contribution of the area of the neck states a brachiocephalic vein involvement, while a bilateral symptomatology most likely involves the SVC [7]. Other rare expressions of CVS/CVO have also been described [8–10]. Ehrie et al. investigated the effect peripheral stenosis has on a simultaneous asymptomatic CVS in arteriovenous fistulas (AVFs), and whether the treatment of the first affects symptom manifestation of the other. In this analysis, the chance of a CVS becoming symptomatic was 4.9%. The second finding was that upper arm AVFs had a significantly higher probability of becoming symptomatic compared to forearm AVFs [11].

**Table 1:** Clinical manifestations of central venous stenosis and occlusion

Clinical Manifestations of Central Venous Stenosis/Occlusion	
Swelling	Ipsilateral arm swelling, pain, tenderness, and/or erythema of the extremity (50% of Cases)
	Ipsilateral breast swelling
	Neck swelling
Visible tributary venous network	
Inadequate dialysis performance	
Less Common	Ipsilateral pleural effusions
	Neurologic manifestations
	Superior Vena Cava syndrome
Based on location of stenosis/occlusion	
Subclavian v	Ipsilateral Arm + Breast
Brachiocephalic v	+ Ipsilateral Neck
SVC and/or Bilateral Brachiocephalic v	+ Bilateral

*SVC* Superior vena cava, *v* vein

## Crossing the Lesion

Crossing a CVS has a higher difficulty index compared to a peripheral venous stenosis due to the potential complications that may occur within the thoracic cavity. Nevertheless, the ability to access the central veins not only from the vascular access or the catheter itself but also from the femoral region and other veins allows additional versatility. Crossing a CVO further increases the complexity of the procedure that is why prior cross-sectional imaging is advisable for proper planning. As in every vascular bed, the shorter the time frame between the occlusion and the intervention the higher the chance of crossing the obstruction. Interestingly, in a systematic review by Andrawos et al. [12] with 655 patients that underwent balloon angioplasty or stenting of a CVS/CVO, failure to cross the lesion was only 3.4%. Wen et al. performed a two-step cluster analysis of 103 patients with CVS/CVO and the patients were divided automatically in two groups based on two distinct phenotypes. One group had the most critical predictors (higher proportion of blunt stump, the presence of side branches, occlusion length > 2 cm, calcification, or organization). Post-intervention primary patency (PIPP) was significantly worse in the group with these characteristics which also had a longer operation time [13].

### Sharp Recanalization

Several endovascular procedures have been proposed for the crossing of chronic CVOs apart from the “traditional” methods that implement the use of different types of wires and catheters and are collectively put under the title “sharp recanalization”. Sharp recanalization is a method of forced crossing a chronic total occlusion [14]. Usually, the procedure is performed with the use of a sharp needle to cross the lesion from one side with a targeting device on the other side, most commonly a snare device or a balloon catheter. Of utmost importance is the use of orthogonal fluoroscopic views to verify positioning. Following crossing of the lesion, inflation of balloons of increasing diameter is performed followed by venograms to assess potential extravasation. A metallic scaffolding may or may not be used based on the angioplasty outcome.

The studies on sharp recanalization involving the intrathoracic central veins are shown in Table 2. Apart from the Chiba needle and the hard back of a wire, other methods include the use of a transseptal needle, the PowerWire Radiofrequency Guidewire (Baylis Medical Company Inc, Montreal, QC, Canada), an excimer laser, the RUPS-100 biopsy set (Rösch-Uchida Transjugular Liver Access Set, Cook Medical, IN, USA) or the Outback catheter (Cordis, CA, USA) [14–31]. Despite the complexity of the procedure, studies describe high percentage of technical success (77.7–100%). In the majority of the cases, stepwise increase in balloon diameter was followed by bare metal stent (BMS) placement and less frequently by covered stent (CS) placement based either on operator’s discretion or the presence of a perforation. Major complications included pericardial perforation with subsequent hemopericardium and hemothorax and were evaluated between 2.3 and 7.4%. Only one study, which included CVOs of different types of patients and central veins from the upper and the lower part of the body, reported the death of a patient after the radiofrequency guidewire perforated the trachea. In the same study authors concluded that longer lesions were characterized by significantly worse outcomes (9 cm vs. 3 cm, *p* = 0.039) [32]. Hence, sharp recanalization is a valid and useful option for crossing a chronically occluded central vein, offering an important additional option to the management of these situations. Moreover, operators need to be aware of the risk of, potentially, serious complications, having covered stents available if needed.

**Table 2:** Studies describing sharp recanalization procedures

Studies describing sharp recanalization procedures									
References	NOP	NOI	Intrathoracic NOI	Method	TS	LL	Complications	AP	Patency
Anil et al. [15]	1	1	1 BC	Outback	100%	N/A	0	B + S	100% @ 6 months
Guimaraes et al. [16]	42	43	43 2 SC, 29 BC, 8 SVC	RF Wire	100%	N/A	1	B + S	95.2% @ 9 months
Iafrati et al. [17]	3	3	3 1 bilateral SC + BC 2 BC	RF Wire	100%	8.2 ± 3.6 cm	0	B + CS	1 angioplasty @ 4 months, Stents patent @ 15 months
Arabi et al. [18]	7	7	7 BC	Transseptal needle	100%	N/A	2	B + CVC placement	
Cohen et al. [14]	39	39	37 3 SC, 3 SC + BC, 2 IJ + BC, 20 BC, 1 BC + SVC, 8 SVC	Chiba needle	95%	4 cm (1–10 cm)	4 minor 2 major	B B + S B + CS	N/A
McDevitt et al. [21]	123	123	59 43 including SC 16 including the SVC	Transseptal needle (87.8% of the cases)	91.5%	N/A	7 minor 1 moderate 3 major	B B + S (65%) B + CS (6.5%)	79.7% @ 28.5 ± 25.0 months
Rambhia et al. [22]	1	1	1 BC	Excimer laser	100%	N/A	0	B + CS	
Keller et al. [19]	18	20	11 5 BC, 5 BC + SVC, 1SC + BC + SVC	RF Wire	80%	4.9 cm (range 0.8–31.7 cm)	1 major	B + S	
Yang et al. [23]	16	16	16 SVC	Guidewire	87.5%	2.81 ± 1.55 cm,	3 major	B + CVC placement	58.33% @ 12 months (CVC)
Gallo et al. [27]	35	36	36 2 SC, 3 SC + BC, 15 BC, 10 SVC + BC, 6 SVC	Chiba needle via an 18-gauge trocar needle	94%	3 cm (range 0.3–5.3 cm)	1 major	B B + CVC	N/A
Yin et al. [24]	16	16	16 2 BC + IJ, 2 BC, 4 BC + SC, 1 BC + SVC	Transseptal needle	81.3%	3 cm (range 2.3–3.6 cm)	0	B B + S	69.2% @ 12 months
Zhao et al. [25]	16	16	16 BC	Chiba Needle	100%	4.4 cm (range 1–8 cm)	2 minor	B + S	81.3% @ 10 months
Chen et al. [26]	25	27	27 3 bilateral SC 18 unilateral BC 6 bilateral BC	Guidewire Chiba needle RUPS-100 biopsy set	92.6%	N/A	4 minor 2 major	B + S B + CS	84% @ 6 months
Wu et al. [28]	14	14	14 SVC	RUPS-100 biopsy set	100%	0.8 ± 0.1 cm	1 minor 1 moderate 2 major	B + S	71.4% @ 12 months
Shen et al. [30]	9	9	9 SVC	Transseptal needle	77.7%	N/A	1 major	N/A	85.7% @ 12 months
Sun et al. [31]	30	30	30 12 SC, 6 SC + BC, 6 BC 2 BC + SVC, 4 SVC	Blunt impingement technique RUPS-100 biopsy set Chiba Needle	100%	3.1 ± 1.5 cm	2 minor	B B + S	53.3% @ 12 months
Huang et al. [29]	1	1	1 SC + BC + IJ	Chiba Needle	100%	N/A	0	B + S	100% @ 8 months

*NOP* Number of patients, *NOI* Number of interventions. *TS* Technical success, *LL* Lesion length, *AP* Adjunctive procedures, *BC* Brachiocephalic vein, *N/A* Not applicable, *B* Balloon angioplasty, *S* Bare metal stent, **@** at, *SC* Subclavian vein, *SVC* Superior vena cava, *RF* Radiofrequency, *CS* Covered stent, *CVC* Central venous catheter, *IJ* Internal jugular vein

## Treating the Lesion

There are two objectives when treating a stenotic lesion in vascular access. The first is the successful mechanical effect of the procedure, which is defined as < 30% residual stenosis comparing the diameter of the treated area with the closest healthy part of the vessel [33]. This is of paramount importance in the case of dialysis patients as there is a pressing need for the patient to return to dialysis and avoid catheter placement. The second objective is extending the time between interventions by slowing down the process of restenosis. To date, this could be achieved with the anti-restenotic effect of paclitaxel-coated balloons (PCB) and the mechanical effect of a metal scaffolding with the use of bare metal stents (BMS) and covered stents (CS), which also constitute bail-out options in case of failed balloon angioplasty.

### Balloon Angioplasty

Based on the KDOQI guidelines, CVS should be treated with plain balloon angioplasty (PBA) and high-pressure balloon (HPB) (> 20 atm) angioplasty as needed [2]. This is mainly due to the lack of evidence when comparing these two categories. However, as described by DePietro et al., HPB is the selection of choice for peripheral stenosis, and the practice of the authors of the current review as well [34]. Ultra-high-pressure balloons (UHPB) (> 30 atm) can also be utilized, but the maximum size of 12 mm is their main limitation in this field. Quality Improvement Guidelines of the Society of Interventional Radiology (SIR), based on limited data, suggested a threshold for success rate of 66%, and a 6-month and a 12-month cumulative patency of 64% and 43%, respectively, for balloon angioplasty without clarifying the type of balloon [33]. Balloon size is also important when treating central veins. In a multicenter retrospective analysis evaluating the use of PCBs for CVS of vascular access, it was found that the only independent factor that positively influenced target lesion primary patency (TLPP) was balloon size [35]. More specifically, an increase by 1 mm in balloon diameter improved TLPP by 29%. It is the authors practice to gradually increase balloon diameter when treating a CVS. A stepwise increase in balloon diameter allows to evaluate the proper balloon diameter taking into consideration not only digital subtraction angiography but also indirect signs like balloon waisting, diminishment of collaterals and patient discomfort.

Elastic recoil is another limiting factor of balloon angioplasty in CVS. This is mainly because the bail-out option of metallic scaffolding may compromise future options and jail the inflow of other central vessels. Rajan et al. [36] observed a 16% of elastic recoil at 15 min following balloon angioplasty of peripheral stenosis in vascular access, without the latter playing a significant role in TLPP in their prospective observational study. A possible tool assisting decision-making could be the use of intravascular ultrasound (IVUS) [37]. Its use can be implemented not only for the evaluation of stenosis but also for the result of balloon angioplasty. Apart from the actual degree of stenosis, IVUS could also provide information regarding the characteristics and potentially its origin, in case where an extrinsic factor is triggering the event. Given the limited amount of data and the increased cost, the device adds to the procedure; its use is still limited.

### Paclitaxel-Coated Balloons

The use of PCBs has been extensively studied in vascular access in both randomized controlled trials (RCT) and cohorts (prospective and retrospective) [38–40]. However, no recommendation is given by the KDOQI guidelines as by the time of their publication the major PCB RCTs where not available [2]. Although no signal regarding mortality has been issued compared to peripheral arterial disease, there is inconsistency regarding their effectiveness [38, 41, 42]. The latter remains a controversial issue as the extensive variability in treatment areas, the different types of vascular access (radiocephalic and brachiocephalic fistulas, arteriovenous grafts, etc.), the different ways that these accesses are created and the different ways that hemodialysis is performed around the world makes the combined analysis of different results difficult, if not impossible [43]. Other factors influencing the outcomes of PCB angioplasty are the time of inflation and the proper prior treatment of the lesion with balloon angioplasty [44]. This was evident in the study by Karnabatidis et al. where the patients with PCB inflation of ≥ 2 min and where vessel preparation took place had independently significantly better results compared to the case that those factors were absent [45].

Central veins, as a treatment area in vascular access, have also been a field where studies have explored the potential use of PCBs (Table 3). There is only one RCT, available by the authors of the current review, including 40 patients and comparing PCBs (Lutonix, Becton Dickinson, Tempe, AZ, USA) to HPB angioplasty alone with PCBs having a significantly better outcome in intervention-free period (179 days vs. 124.5 days, *p* = 0.026) [46]. In an all-comers prospective registry by Karnabatidis et al. which included 392 lesions treated with a PCB (Lutonix) in vascular access, 20 of them were a CVS. TLPP was 65% at 6 months for this treatment area [45]. A prospective longitudinal comparison between balloon angioplasty (first treatment) and PCB [second treatment (Elutax, Aachen Resonance, Aachen, Germany)] in 18 patients was performed by Cakir et al. Results were significantly better for the PCB group (intervention-free period: 109 vs. 238.5 days, *p* < 0.001) [47]. A retrospective study of 30 patients by Chong et al. longitudinally compared the outcomes of PCB angioplasty (Lutonix) to balloon angioplasty on the same treatment area. Although PCBs had a longer intervention-free period, the difference did not reach significance (164 vs. 140 days, *p* = 0.257) [48]. In the same setting Hongsakul et al. also longitudinally compared PCBs in patients with early recurrence (< 30 days) following balloon angioplasty. Investigators used parallel PCBs (IN.PACT, Medtronic, MN, USA) to match the vessel diameter in this study due to the 12-mm-diameter limitation of the available balloons. TLPP was 93.8% at 6 months and 31.2% at 12 months [49]. A multicenter European retrospective analysis of PCB use (Lutonix, IN.PACT, Elutax) in 86 cases had a clinically assessed intervention-free period of 62.7% at 6 months and 34.6% at 12 months [35]. Finally, Massmann et al. used a custom-made up to 14-mm PCB (Elutax) in their retrospective analysis of 10 patients showing significantly better results compared to plain balloon angioplasty. In these 10 patients, authors also included axillary vein stenosis [50].

**Table 3:** Paclitaxel-coated balloons (PCBs)

Paclitaxel-Coated Balloons				
Devices used in the studies				
Device	Company	Dose (μg/mm^2^)	Excipient	Maximum Diameter
Lutonix	Becton Dickinson, Franklin Lakes, NJ, USA	2	Polysorbate/Sorbitol	12 mm
IN.PACT	Medtronic, Dublin, Ireland	3.5	Urea	12 mm
Elutax-SV	Aachen Resonance, Aachen, Germany	2.2	No Excipient/Dextran coating	14 mm

Studies							
References	Type	Device	Country	Pts with PCB	VA Circuit	Results	Comments
Massmann et al. [50]	SC-DA-RS	Elutax-SV	Germany	10	AVF	SSD @ 9 m	Included axillary veins
Kitrou et al. [40]	SC-RCT	Lutonix	Greece	20	AVF, AVG	SSD @ 6 m	
Hongsakul et al. [49]	SC-SA-RS	IN.PACT	Thailand	16	AVF, AVG	TLPP @ 6 m (93.8%), 12 m (31.2%)	Stenoses previously treated with balloon angioplasty and had early recurrence (< 3 months)
Karnabatidis et al. [45]*	MS-SA-PS	Lutonix	Global (Exc. USA)	20	AVF, AVG	TLPP @ 6 m 65%	Subgroup of patients from an all-comers study (320 pts in total)
Kitrou et al. [35]	MS-SA-RS	Lutonix, IN.PACT, Elutax-SV	European	86	AVF, AVG	IFP @ 6 m (62.7%)	
Chong et al. [48]	SC-DA-RS	Lutonix	Singapore	30	AVF, AVG	NSD @ 6 m	Longitudinal comparison with PBA
Cakir et al. [47]	SC-DA-PS	Elutax-SV	Turkey	18	AVF	MP, SSD	Longitudinal comparison with PBA

Table showing available studies of PCBs including those where only a cohort had a central venous stenosis (with *)

*AVF* Arteriovenous fistula, *AVG* Arteriovenous graft, *DA* Double-Arm, *LLL* Late lumen loss, *MS* Multicenter, *NSD* Non-statistically significant difference, *PBA* Plain balloon angioplasty, *PCB* Paclitaxel-coated balloon, *RCT* Randomized controlled trial, *RS* Retrospective study, *SA* Single-arm, *SC* Single-center, *SSD* Statistically significant difference, *TLPP* Target lesion primary patency, *MP* Median patency

It is becoming apparent that primary patency rates appearing in the above-mentioned studies are numerically equal, and in some cases better, compared to the suggested performance goal of SIR quality improvement guidelines for the cumulative patency rates of balloon angioplasty and one can argue that there is a benefit in their use. In RCTs performed in outflow veins of vascular access, one can see that the effect is amplified with proper vessel preparation and time of balloon inflation of more than 2 min. In fact, the study by Lookstein et al. on peripheral stenosis in arteriovenous fistulas had an inflation time of 3 min and showed a significant benefit for PCB over balloon angioplasty even at 3 years (43.1% vs. 28.6%) [51]. A limitation of PCBs in the treatment of CVS is their size reaching 12 mm. Although a predilation is suggested to achieve better outcomes, initial studies on PCBs, when the maximum balloon diameter was 7 mm, have shown that post-dilation of a lesion after PCB angioplasty also showed significantly better results compared to balloon angioplasty [52]. With no evidence to support it, this is the authors’ current practice for CVS that need a final balloon dilatation of over 12 mm.

### Bare Metal Stents

The aggressive nature of neointimal hyperplasia present in CVS limits the use of BMS in vascular access in general and in central vein in particular, as tissue emerges through the bare strut area. Hence, the recent guidelines suggest avoiding their use [2]. A meta-analysis by Wu et al. of 8 studies showed no benefit between balloon angioplasty and BMS in primary patency, but a significantly higher assisted primary patency was observed in favor of balloon angioplasty at 2 years [53].

As previously discussed however, BMS has been extensively used to support the results of sharp recanalization. Additionally, the use of self-expanding stents in this location, needing an over-dilation, makes BMSs the only available option based on the size options compared to CS. Akkakrisee et al. compared BMS to dedicated venous stents (Sinus and Sinus XL venous stent Optimed, Ettlingen, Germany) for the treatment of central venous stenosis. In this retrospective study of 77 patients, 34 received a dedicated venous stent. Primary patency was better with dedicated venous stents vs. bare metal stents at 12 months (61.8% vs. 32.6%; *p* = 0.008) [54].

### Covered Stents

Covered stents have established their role for the treatment of the dysfunctional vascular access mainly for the treatment of the venous anastomosis of grafts and the in-stent restenotic lesions [55]. KDOQI guidelines also advice their use for CVS as instructed in peripheral stenosis in vascular access [2]. SIR guidelines do not differentiate the suggested thresholds between BMS and CS for the treatment of CVS [33]. Size availability is the main limitation of their suitability for CVS treatment although new CS are available to sizes up to 16 mm [56]. Moreover, the possibility of jailing other central veins is another limitation. The available CSs and the studies on them are presented in Table 4.

**Table 4:** Covered stents (CSs)

Covered Stents			
Devices used in the studies			
Device	Company	Maximum Diameter	Maximum Sheath Size
Viabahn	W.L Gore and Associates, AZ, USA	13 mm	12 Fr
Fluency	Becton Dickinson, Tempe, AZ, USA	13.5 mm	10 Fr
Wrapsody	Merit Medical UT, USA	16 mm	14 Fr
Excluder AAA Leg	W.L Gore and Associates, AZ, USA	16 mm	12 Fr

Studies								
Study	Type	Device	Country	Pts	CS used	VA Circuit	Results	Comments
Anaya-Ayala et al. [58]	SC-SA-RS	Viabahn 24 pts Fluency 1 pt	USA	25	25	AVF, AVG	TS: 100% TLPP: 56% @ 12 m	20/25 pts with functional vascular access
Jones et al. [57]	SC-SA-RS	Viabahn	UK	30	42	AVF	TS: 100% TLPP: 45% @ 24 m	Bail-out procedures
Verstandig et al. [60]	SC-SA-RS	Viabahn 29 pts Fluency 23 pts	Israel	52	57	AVF, AVG	TS: 100% TLPP: 28% @ 36 m	
Boutrous et al. [59]	SC-SA-RS	Viabahn	USA	29	> 29	AVF, AVG	TS: 100% TLPP: 80% @ 24 m	Longer stents were significantly associated with restensosis
Chen et al. [61]	SC-SA-RS	Excluder	Taiwan	60	60	AVF, AVG	TS: 100% TLPP: 80.25% @ 24 m	20% occlusions
Gilbert et al. [56]*	MC-SA-PS	Wrapsody	Global	11	> 11	AVF, AVG	TS: 100% TLPP: 100% @ 6 m	Subgroup pf patients from a larger registry

Table showing available studies of CSs including those where only a cohort had a central venous stenosis (with *)

*SC* Single-center, *SA* Single-arm, *RS* Retrospective study, *@* at, *MC* Multicenter, *PS* Prospective study, *TS* Technical success, *TLPP* Target lesion primary patency

There are three studies available for the use of the Viabahn (W.L Gore and Associates, AZ, USA) CS for the treatment of CVS. Jones et al. evaluated the use of 42 CSs as a bail-out option in 30 patients in their retrospective analysis. Mean follow-up was 702 days, and TLPP was 67% and 45% at 12 and 24 months, respectively. Patients with a previous intervention (balloon angioplasty or CS) had significantly worse results compared to de novo lesions [57]. Anaya-Ayala et al. included 25 patients [in one case a Fluency CS was used (Becton Dickinson, Tempe, AZ, USA)]. Two cases were thrombosed within 30 days and another at 3 months with TLPP been 56% at 12 months [58]. The third retrospective analysis from Boutrous et al. studied 29 patients. Patency rates were 91.7% and 80% at 12 and 24 months, respectively. Authors reported that longer stents had worse results [59]. In a retrospective study of 52 patients, Verstandig et al. tested the Viabahn (29 patients, 30 CS) and the Fluency (23 patients, 27 CS). TLPP was 60% and 40% at 6 and 12 months, respectively [60]. In a pilot single-arm prospective registry by Gilbert et al. using the Wrapsody CS (Merit Medical UT, USA) for the treatment of outflow stenosis in vascular access, out of the 46 patients included, 11 had a CS inserted to their central veins. TLPP was 100% at 12 months [56]. Finally, a very interesting study from Chen et al. used the contralateral limb of the Gore Excluder AAA leg stent graft or iliac extender endoprosthesis (W.L Gore and Associates, AZ, USA) for the treatment of CVS. In their prospective registry authors included 60 patients. Antegrade access from the arm vein was gained in 56 cases, while in 3 cases the femoral access was used and the jugular in one. Follow-up period was 21.6 months. Target site primary patency was 88.3% and 80.3% at 12 and 24 months, respectively [61].

## Conclusion

Central venous stenosis and chronic occlusions remain a significant challenge in vascular access treatment. Endovascular procedures are the primary treatment option as novel techniques, and devices allowed the negotiation of both stenosed and completely occluded central veins. The gold standard treatment remains the use of balloon angioplasty (with HPB angioplasty as needed), while PCBs seem to forestall restenosis and extend the time interval to re-intervention. Covered stent use is a valuable bail-out option. Although not improving results compared to angioplasty and suggested to have inferior results to covered stents, bare metal stents can still be considered a bail-out option for sizes not available in covered stents.

## References

[1] Quencer KB, Arici M (2015). Arteriovenous fistulas and their characteristic sites of stenosis. AJR Am J Roentgenol.

[2] Lok CE (2020). KDOQI clinical practice guideline for vascular access: 2019 update. Am J Kidney Dis.

[3] Schillinger F (1991). Post catheterisation vein stenosis in haemodialysis: comparative angiographic study of 50 subclavian and 50 internal jugular accesses. Nephrol Dial Transpl.

[4] Yevzlin AS (2008). Hemodialysis catheter-associated central venous stenosis. Semin Dial.

[5] Adwaney A (2019). Central venous stenosis, access outcome and survival in patients undergoing maintenance hemodialysis. Clin J Am Soc Nephrol.

[6] Trerotola SO (2015). Central venous stenosis is more often symptomatic in hemodialysis patients with grafts compared with fistulas. J Vasc Interv Radiol.

[7] Kundu S (2010). Review of central venous disease in hemodialysis patients. J Vasc Interv Radiol.

[8] Mochida Y (2021). Angiectasia of the parietal pleura in a hemodialysis patient with central venous stenosis and bloody pleural effusion: a case report. CEN Case Rep.

[9] Kang H, Park ST (2022). Resolved cerebral venous hypertension after angioplasty of central venous stenosis in a hemodialysis patient: a case report. Taehan Yongsang Uihakhoe Chi.

[10] de Freitas D, Moss J (2020). Innominate vein stenosis causing raised intracranial pressure and blindness. J Vasc Surg Cases Innov Tech.

[11] Ehrie JM (2017). Unmasking of previously asymptomatic central venous stenosis following percutaneous transluminal angioplasty of hemodialysis access. J Vasc Interv Radiol.

[12] Andrawos A, Saeed H, Delaney C (2021). A systematic review of venoplasty versus stenting for the treatment of central vein obstruction in ipsilateral hemodialysis access. J Vasc Surg Venous Lymphat Disord.

[13] Wen C (2022). Clinical implications of phenotypes of hemodialysis patients with central venous occlusion or central venous stenosis defined by cluster analysis. Front Cardiovasc Med.

[14] Cohen EI, Beck C, Garcia J, Muller R, Bang HJ, Horton KM, Hakki F (2018). Success rate and complications of sharp recanalization for treatment of central venous occlusions. Cardiovasc Intervent Radiol.

[15] Anil G, Taneja M (2010). Revascularization of an occluded brachiocephalic vein using Outback-LTD re-entry catheter. J Vasc Surg.

[16] Guimaraes M (2012). Radiofrequency wire for the recanalization of central vein occlusions that have failed conventional endovascular techniques. J Vasc Interv Radiol.

[17] Iafrati M, Maloney S, Halin N (2012). Radiofrequency thermal wire is a useful adjunct to treat chronic central venous occlusions. J Vasc Surg.

[18] Arabi M (2016). Sharp central venous recanalization in hemodialysis patients: a single-institution experience. Cardiovasc Intervent Radiol.

[19] Keller EJ (2018). Single-center retrospective review of radiofrequency wire recanalization of refractory central venous occlusions. J Vasc Interv Radiol.

[20] Majdalany BS (2019). Radiofrequency wire recanalization of chronically occluded venous stents: a retrospective, single-center experience in 15 patients. Cardiovasc Intervent Radiol.

[21] McDevitt JL, Srinivasa RN, Gemmete JJ, Hage AN, Srinivasa RN, Bundy JJ, Chick JFB (2019). Approach, technical success, complications, and stent patency of sharp recanalization for the treatment of chronic venous occlusive disease: experience in 123 patients. Cardiovasc Intervent Radiol.

[22] Rambhia S, Janko M, Hacker RI (2018). Laser recanalization of central venous occlusion to salvage a threatened arteriovenous fistula. Ann Vasc Surg.

[23] Yang L (2020). The feasibility and safety of sharp recanalization for superior vena cava occlusion in hemodialysis patients: a retrospective cohort study. Hemodial Int.

[24] Yin X (2020). Efficacy and safety of recanalization with transseptal needle for chronic total occlusion of the brachiocephalic vein in hemodialysis patients. Ann Transl Med.

[25] Zhao Y (2020). Sharp recanalization of the brachiocephalic vein occlusion through the external jugular vein in hemodialysis patients. Ann Transl Med.

[26] Chen B, Lin R, Dai H, Li N, Tang K, Yang J, Huang Y (2022). Sharp recanalization for treatment of central venous occlusive disease in hemodialysis patients. J Vasc Surg Venous Lymphat Disord.

[27] Gallo CJR (2021). Sharp recanalization of chronic central venous occlusions of the thorax using a steerable coaxial needle technique from a supraclavicular approach. Cardiovasc Intervent Radiol.

[28] Wu XW (2021). Effectiveness of sharp recanalization of superior vena cava-right atrium junction occlusion. World J Clin Cases.

[29] Huang XR (2022). Percutaneous superior vena cava puncture successful recanalization of a long-segment, angled central venous occlusion: a case report. Ann Palliat Med.

[30] Shen X, Zhao Q, Salerno S, Cui T (2022). Sharp recanalization with transseptal needle for superior vena cava occlusion: a retrospective single-center analysis. Asian J Surg.

[31] Sun JB (2022). The efficacy and safety of blunt impingement followed by a sharp recanalization technique in hemodialysis patients with refractory central vein occlusion: a single-center experience. Ann Transl Med.

[32] Sivananthan G (2015). Safety and efficacy of radiofrequency wire recanalization of chronic central venous occlusions. J Vasc Access.

[33] Dariushnia SR (2016). Quality improvement guidelines for percutaneous image-guided management of the thrombosed or dysfunctional dialysis circuit. J Vasc Interv Radiol.

[34] De Pietro DM, Trerotola SO. Choosing the right treatment for the right lesion, part I: a narrative review of the role of plain balloon angioplasty in dialysis access maintenance*.* Cardiovascular Diagnosis Therapy, 2022. 10.21037/cdt-22-375 PMC9971312 36864950

[35] Kitrou PM, Steinke T, El Hage R, Ponce P, Lucatelli P, Katsanos K, Spiliopoulos S, Spinelli A, Bisdas T, Stavroulakis K, Jaffer O, Mallios A, Zilahi de Gyurgyokai S, Cancellieri R, Coscas R, Karnabatidis D (2021). Paclitaxel-coated balloons for the treatment of symptomatic central venous stenosis in vascular access: results from a european, multicenter, single-arm retrospective analysis. J Endovasc Ther.

[36] Rajan DK (2016). Elastic recoil after balloon angioplasty in hemodialysis accesses: Does it actually occur and is it clinically relevant?. Radiology.

[37] de Graaf R (2016). The value of intravascular ultrasound in the treatment of central venous obstructions in hemodialysis patients. J Vasc Access.

[38] Lookstein RA, Haruguchi H, Holden A (2021). Drug-coated balloons for dysfunctional dialysis arteriovenous fistulas. Reply N Engl J Med.

[39] Trerotola SO (2018). Drug coated balloon angioplasty in failing AV fistulas: a randomized controlled trial. Clin J Am Soc Nephrol.

[40] Kitrou PM (2017). Paclitaxel-coated balloons for the treatment of dysfunctional dialysis access. Results from a single-center, retrospective analysis. Cardiovasc Intervent Radiol.

[41] Trerotola SO (2020). The lutonix av randomized trial of paclitaxel-coated balloons in arteriovenous fistula stenosis: 2-year results and subgroup analysis. J Vasc Interv Radiol.

[42] Katsanos K, Kitrou P, Spiliopoulos S (2021). The rollercoaster of paclitaxel in the lower limbs and skeletons in the closet: an opinion review. J Vasc Interv Radiol.

[43] Kitrou P (2022). Drug-coated balloons for the dysfunctional vascular access: an evidence-based road map to treatment and the existing obstacles. Semin Intervent Radiol.

[44] Karnabatidis D, Kitrou P (2017). Drug eluting balloons for resistant arteriovenous dialysis access stenosis. J Vasc Access.

[45] Karnabatidis D (2021). A multicenter global registry of paclitaxel drug-coated balloon in dysfunctional arteriovenous fistulae and grafts: 6-month results. J Vasc Interv Radiol.

[46] Kitrou PM (2017). Paclitaxel-coated balloons for the treatment of symptomatic central venous stenosis in dialysis access: results from a randomized controlled trial. J Vasc Interv Radiol.

[47] Cakir S, Guzelbey T, Mutlu IN, Kilickesmez O (2022). Comparison of functional patency rates between paclitaxel-eluting versus plain balloon angioplasty in hemodialysis patients with central venous stenosis: an intra-individual comparison study. Ther Apher Dial.

[48] Chong TT (2020). Use of paclitaxel coated drug eluting technology to improve central vein patency for haemodialysis access circuits: Any benefit?. Vasc Specialist Int.

[49] Hongsakul K (2018). Paclitaxel-coated balloon angioplasty for early restenosis of central veins in hemodialysis patients: a single center initial experience. Korean J Radiol.

[50] Massmann A (2015). Paclitaxel-coated balloon angioplasty for symptomatic central vein restenosis in patients with hemodialysis fistulas. J Endovasc Ther.

[51] Holden A. The IN.PACT AV Study*.* EndoVascular Access Meeting, 2022.

[52] Kitrou PM (2015). Paclitaxel-coated versus plain balloon angioplasty for dysfunctional arteriovenous fistulae: one-year results of a prospective randomized controlled trial. J Vasc Interv Radiol.

[53] Wu TY (2020). Comparison of percutaneous transluminal angioplasty with stenting for treatment of central venous stenosis or occlusion in hemodialysis patients: a systematic review and meta-analysis. Cardiovasc Intervent Radiol.

[54] Akkakrisee S, Hongsakul K (2022). Venous stent versus conventional stent for the treatment of central vein obstruction in hemodialysis patients: a retrospective study. Acta Radiol.

[55] Haskal ZJ (2010). Stent graft versus balloon angioplasty for failing dialysis-access grafts. N Engl J Med.

[56] Gilbert J (2021). First clinical results of the merit WRAPSODY™ cell-impermeable endoprosthesis for treatment of access circuit stenosis in haemodialysis patients. Cardiovasc Intervent Radiol.

[57] Jones RG, Willis AP, Jones C, McCafferty IJ, Riley PL (2011). Long-term results of stent-graft placement to treat central venous stenosis and occlusion in hemodialysis patients with arteriovenous fistulas. J Vasc Interv Radiol.

[58] Anaya-Ayala JE, Smolock CJ, Colvard BD, Naoum JJ, Bismuth J, Lumsden AB, Davies MG, Peden EK (2011). Efficacy of covered stent placement for central venous occlusive disease in hemodialysis patients. J Vasc Surg.

[59] Boutrous ML, Alvarez AC, Okoye OT, Laws JC, Jacobs DL, Smeds MR (2019). Stent-graft length is associated with decreased patency in treatment of central venous stenosis in hemodialysis patients. Ann Vasc Surg.

[60] Verstandig AG, Berelowitz D, Zaghal I, Goldin I, Olsha O, Shamieh B, Shraibman V, Shemesh D (2013). Stent grafts for central venous occlusive disease in patients with ipsilateral hemodialysis access. J Vasc Interv Radiol.

[61] Chen YY, Wu CK, Lin CH (2020). Outcomes of the Gore Excluder abdominal aortic aneurysm leg endoprosthesis for treatment of central vein stenosis or occlusion in patients with chronic hemodialysis. J Vasc Surg Venous Lymphat Disord.

